# Long non-coding RNA Opa interacting protein 5-antisense RNA 1 binds to micorRNA-34a to upregulate oncogenic PD-L1 in non-small cell lung cancer

**DOI:** 10.1080/21655979.2022.2036904

**Published:** 2022-04-12

**Authors:** Xinwei Qiao, Feng Zhao

**Affiliations:** Department of Thoracic Surgery, Union Hospital, Tongji Medical College, Huazhong University of Science and Technology, Wuhan City, Hubei Province, China

**Keywords:** OIP5-AS1, proliferation, miR-34a, non-small cell lung cancer, PD-L1

## Abstract

Long non-coding RNA (lncRNA) OPA-interacting protein 5 antisense transcript 1 (OIP5-AS1) plays an oncogenic role in several types of cancer, but whether it is involved in non-small-cell lung cancer (NSCLC) is unclear. Our preliminary sequencing analysis revealed the upregulation of OIP5-AS1 in NSCLC. In this study, gene expression levels were analyzed by RT-qPCR. RNA-RNA pull-down assay was applied to detect direct interactions between RNAs. Overexpression assays were performed to explore the relationship between miR-34a and OIP5-AS1. CCK-8 assay and colony formation assay were applied to evaluate cell proliferation. In NSCLC cells (H23), overexpression of OIP5-AS1 increased the expression levels of programmed death-ligand 1 (PD-L1). In addition, inhibition of OIP5-AS1 and overexpression of miR-34a decreased the expression levels of PD-L1, and miR-34a significantly blocked the role of overexpression of OIP5-AS1. Overexpression of OIP5-AS1 and PD-L1 promoted H23 and H22 cells proliferation, while silencing of miR-34a and OIP5-AS1 played opposite roles and eliminated the effects of overexpression of OIP5-AS1 on cell proliferation. Therefore, OIP5-AS1 was upregulated to enhance the expression of oncogenic PD-L1 by sponging miR-34a in NSCLC, leading to promoted NSCLC cell proliferation. Our study also demonstrated that OIP5-AS1 was upregulated while miR-34a was downregulated in NSCLC.

## Introduction

Lung cancer is responsible for about 11.4% of all cancer cases and causes 18.0% of all cancer-related mortalities [1,2]. In some Asian countries, such as Korea, the number of deaths caused by lung cancer even exceeds the sum of the deaths caused by stomach, colorectal, and pancreatic cancer [3]. Smoking has been recognized as the most dangerous risk factor for lung cancer [4], while never-smokers can still suffer from all types of lung cancer [5]. Lung cancer can be classified into different clinical types, including non-small cell lung cancer (NSCLC), undifferentiated large cell lung cancer, squamous cell lung cancer and lung adenocarcinoma. Among these, NSCLC has the highest incidence rate and is the most lethal one. Other factors, such as genetic factors, should also be investigated when studying the pathogenesis of lung cancer [6].

Programmed death-ligand 1 (PD-L1) is a transmembrane protein with critical roles in regulating the immune system [7]. In cancer biology, PD-L1 can be regulated by oncogenic miRNAs, such as miR-940 [8], and tumor suppressive miRNAs, such as miR-34a [9], to impact cancer cell behaviors including proliferation and migration. Studies have demonstrated the prognostic value of PD-L1 in lung cancer [10–12]. In effect, PD-L1 has been considered as a new target of cancer immunotherapy [13].

Long non-coding RNAs (lncRNAs) are involved in diverse biological processes [14]. Different from protein-coding genes, lncRNAs are mainly expressed in some types of cells and play specific roles in cellular activities, especially the regulation of cancer cell behaviors [15,16]. Recently studies have shown that OPA-interacting protein 5 antisense transcript 1 (OIP5-AS1) is involved in many cancers [17]. For instance, OIP5-AS1 positively regulates OIP5 to promote bladder cancer [18]. Moreover, overexpression of OIP5-AS1 contributes to poor survival of bladder cancer [19]. In colorectal cancer, overexpression of OIP5-AS1 mediates the development of radioresistance of cancer cells through the interaction with miR-369-3p by targeting DYRK1A and leads to the failure of treatment [20]. However, there are few investigations on the role of OIP5-AS1 in lung cancer. One study reported that overexpression of OIP5-AS1 promoted lung cancer cell proliferation by suppressing miR-378a-3p [21]. As an important player in cancer progression, OIP5-AS1 has not been fully studied in lung cancer. Therefore, more studies on the role of OIP5-AS1 are urgently needed. We predicted that OIP5-AS1 could bind to miR-34a, which plays a tumor-suppressive role in lung cancer [22]. OIP5-AS1 and miR-34a may interact with each other to participate in NSCLC. We then explore the crosstalk among OIP5-AS1, PD-L1 and miR-34a in NSCLC.

## Materials and methods

### Patients and specimens

A total of 68 NSCLC patients (adenocarcinoma, sex: 48 males and 20 females; age range: 37 to 65 years old; mean age: 50.7 ± 5.1 years old) were included in this study. These patients were admitted at the Union Hospital, Tongji Medical College, Huazhong University of Science and Technology from August 2016 to January 2019. All patients were educated with the experimental principle of this study and signed the informed consent. According to AJCC staging criteria, 16, 20 and 32 patients were classified into clinical stage II–IV, respectively. Patients received the surgical resection of the primary tumors, which were dissected by experienced histopathologists to separate non-tumor lung tissues from NSCLC tissues. Tissue samples were kept in liquid nitrogen before use. This study was approved by the Ethics Committee of Union Hospital, Tongji Medical College, Huazhong University of Science and Technology (TJ-2017-0203). The clinical features of the patients are listed in Table 1.

**Table 1.** Correlations between OIP5-AS1 and clinical characteristics of NSCLC patients

Characteristics	Total number	OIP-5-AS1 expression		P value
		High (n = 34)	Low (n = 34)	
Gender				0.326
Male	33	17	16	
Female	35	17	18	
Age				0.332
<60	31	15	16	
≥60	37	19	18	
Smoking history				0.113
Yes	37	17	20	
No	31	17	14	
TNM staging				0.03
II	16	4	12	
III	20	6	14	
IV	32	24	8	
Tumor size (cm)				0.02
<3	18	14	4	
≥3	50	20	30	

### NSCLC cells and transient transfections

Human NSCLC (adenocarcinoma) cell lines H522 and H23 were obtained from American Type Culture Collection (ATCC). PcDNA3.1-OIP5-AS1 and empty pcDNA3.1 vector, pcDNA3.1-PD-L1 expression vector (Invitrogen, Shanghai, China), miR-34a mimic (5’-UGGCAGUGUCUUAGCUGGUUGU-3’), negative control (NC) miRNA (5’-UGGUGUACCACGUGUAGCUAGU-3’), siRNA NC (5’-GGUAGUCAUUUGCAGUGUGGACA-3’) and OIP5-AS1 siRNA (5’-UGUUAGCUGGAUGACUGGAAUCC-3’) were used to transfect cells using lipofectamine 2000.

### Dual luciferase reporter assay

Dual-luciferase reporter assay was performed as previously described [23]. OIP5-AS1 cDNA (full length) was inserted into pmirGLO. OIP5-AS1 vector was co-transfected with miR-34a mimic or NC-miRNA into 10^6^ cells using aforementioned methods. Dual-luciferase reporter gene assay system was used.

### RNA Pull-down assay

MiR-34a, mutant form of miR-34a (miR-34a (mut)) and control RNA were labeled with biotin and transfected into cells. After cell lysis at 48 h later, magnetic beads were used to isolate complex. The expression of OIP5-AS1 was detected by RT-qPCR.

### RNA extraction

H23 cells were collected and counted. NSCLC tissues non-tumor tissues, as well as cells were used for RNA extraction with Ribozol. RNA samples were precipitated and then washed using 80% ethanol to harvest miRNAs. RNA quality was evaluated by Urea-PAGE gel electrophoresis.

### RT-qPCR

Trizol reagent was used to extracted total RNAs from NSCLC cell lines or tissues. Synthesis of cDNA was performed through reverse transcription using AMV Reverse Transcriptase kit (Sangon, Shanghai, China) with total RNAs as template. qPCRs were performed to detect the expression of PD-L1, OIP5-AS1 and miR-34a with 18S rRNA and U6 as internal controls. Sequences of primers were: 5′-TGCGAAGATGGCGGAGTAAG-3′ (forward) and 5′-TAGTTCCTCTCCTCTGGCCG-3′ (reverse) for OIP5-AS1; 5′-TATGGTGGTGCCGACTACA-3′ (forward) and 5’-TGGCTCCCAGAATTACCAA-3′ (reverse) for PD-L1; 5′-CTCAGACACCATGGGGAAGGTGA-3’ (forward) and 5′-ATGATCTTGAGGCTGTTGTCATA-3′ (reverse) for GAPDH; 5′-TGGCAGTGTCTTAGCTGGTTGT-3′ (forward) and 5′-GTGTCGTGGAGTCGGCAATTGC-3′ (reverse) for miR-34a; 5′-GCTTCGGCAGCACATATACTAAAAT-3′ (forward) and 5′-CGCTTCACGAATTTGCGTGTCAT-3′ (reverse) for U6. Relative expression levels were calculated via the 2^−∆∆Cq^ method [24].

### Cell proliferation analysis

CCK-8 kit was used for cell proliferation analysis. H522 and H23 cells were collected and counted. Then 4 × 10^4^ cells were dissolved in 1 ml RPMI-1640 medium (10% FBS) to make suspension of single cells. Next, cell suspension (100 µl) was added into each well of 96-well plate, which was kept in a 5% CO_2_ incubator at 37°C with 95% humidity to culture the cells. To monitor cell proliferation, CCK-8 solution (10 µl) was added into each well. OD values at 450 nm were determined. To form colony, cells were transferred to 10 cm dish. Colonies were counted 2 weeks later.

### Western blot

H23 cells were collected and counted. Then 5 × 10^5^ cells were dissolved in 1 ml RIPA for protein isolation. BCA kit (Sangon, Shanghai, China) was used for quantification, followed by protein denaturing in boiled water. All protein samples were subjected to electrophoresis using 10% SDS-PAGE gels. Protein samples were then transferred to PVDF membranes and then membranes were blocked in PBS at room temperature for 1.5 h. After that, incubation with primary antibodies of PD-L1 (1:1,200, ab213524, Abcam, Cambridge, UK) and GAPDH (1:1,200, ab37168, Abcam, Cambridge, UK) was performed at 4°C overnight. Following that, goat secondary antibody of HRP (IgG) (1:1,000; ab6721; Abcam, Cambridge, UK) was used to incubate with membranes at 24°C for 2 h. After using ECL for signal production, signals were processed using Image J v1.48 software.

### Immunohistochemistry (IHC)

Immunohistochemistry staining for PD-L1 was performed on 5 randomly selected NSCLC and non-tumor tissues through conventional methods. The primary antibody in our IHC study was rabbit polyclonal primary antibodies of PD-L1.

### Statistical analysis

The GraphPad Prism 7 (GraphPad Software, US) software was used for statistical analysis. Datasets were compared by Student’s t test. *P* < 0.05 was considered as statistically significant.

## Results

### The expression of OIP5-AS1 and PD-L1 in NSCLC

**Figure 1.**
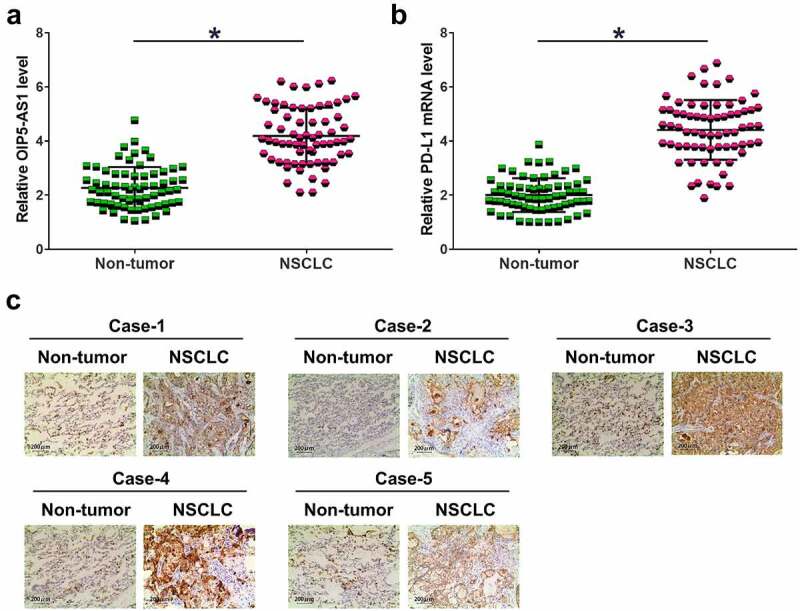
The expression of OIP5-AS1 and PD-L1 in NSCLC. The expression levels of OIP5-AS1 mRNA (a) and PD-L1 mRNA (b) were both elevated in NSCLC tumor and non-tumor samples. *, *p* < 0.05. IHC was used to detect PD-L1 protein in 5 pairs of NSCLC tumor tissues and non-tumor tissues from 5 patients (c). In all 5 cases, the expression of PD-L1 protein was much stronger in NSCLC tumors compared to that in normal non-tumor tissues.

Gene expression analysis is the first step to elucidate gene function. To this end, RT-qPCR was applied for to analyze the expression of OIP5-AS1 and PD-L1 in NSCLC and non-tumor tissue samples. The results showed that the expression levels of OIP5-AS1 (Figure 1(a)) and PD-L1 (Figure 1(b)) were increased in NSCLC (*p* < 0.05). IHC was used to detect the expression of PD-L1 protein in 5 pairs of NSCLC and non-tumor tissues. Cases 1, 2 and 5 were NSCLC patients at stage II, and cases 3 and 4 were patients at stage III/IV. It was observed that PD-L1 signal was much stronger in NSCLC tissues (Figure 1(c)).

### OIP5-AS1 and PD-L1 were positively correlated in NSCLC

**Figure 2.**
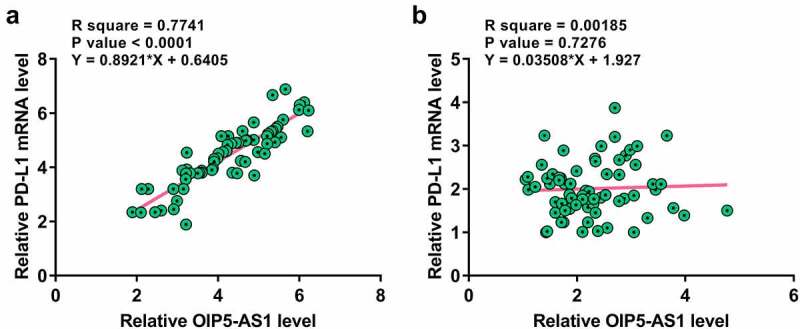
OIP5-AS1 and PD-L1 were positively correlated in NSCLC. Correlations between OIP5-AS1 and PD-L1 mRNA expression in NSCLC tissues (a) and non-tumor tissues (b) were studied with Pearson’s correlation coefficient. *p* < 0.01 for tumor samples; *p* = 0.7276 for non-tumor samples.

Correlations suggest interaction. Therefore, the correlation between OIP5-AS1 and PD-L1 was analyzed with Pearson’s correlation coefficient. The results showed that OIP5-AS1 was positively correlated with PD-L1 across NSCLC tissues (Figure 2(a)). However, no obvious correlation was observed in non-tumor tissues (Figure 2(b)). Therefore, OIP5-AS1 and PD-L1 may interact with each other in NSCLC.

### The expression levels of OIP5-AS1 and PD-L1 were increased with the development of clinical stages

**Figure 3.**
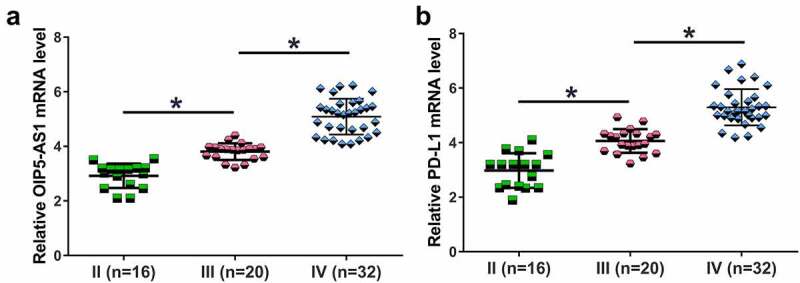
Both OIP5-AS1 and PD-L1 mRNAs were elevated with the increase of clinical stages. The 68 NSCLC patients were classified into three clinical stages according to the severity and clinical standard. There were 16, 20 and 32 patients subgrouped at clinical stage II–IV respectively. The expression levels of OIP5-AS1 (a) and PD-L1 (b) were both elevated with the increase of clinical stages. **, *p* < 0.01.

Cancer stage significantly affects the survival of patients. The 68 NSCLC patients included 16, 20 and 32 patients at clinical stage II–IV, respectively. The expression levels of OIP5-AS1 (Figure 3(a)) and PD-L1 (Figure 3(b)) increased with the development of clinical stages (*p* < 0.01). These results suggested that increased expression levels of OIP5-AS1 and PD-L1 might promote the progression of NSCLC.

### OIP5-AS1 upregulated PD-L1 in H23 cells through regulating miR-34a

**Figure 4.**
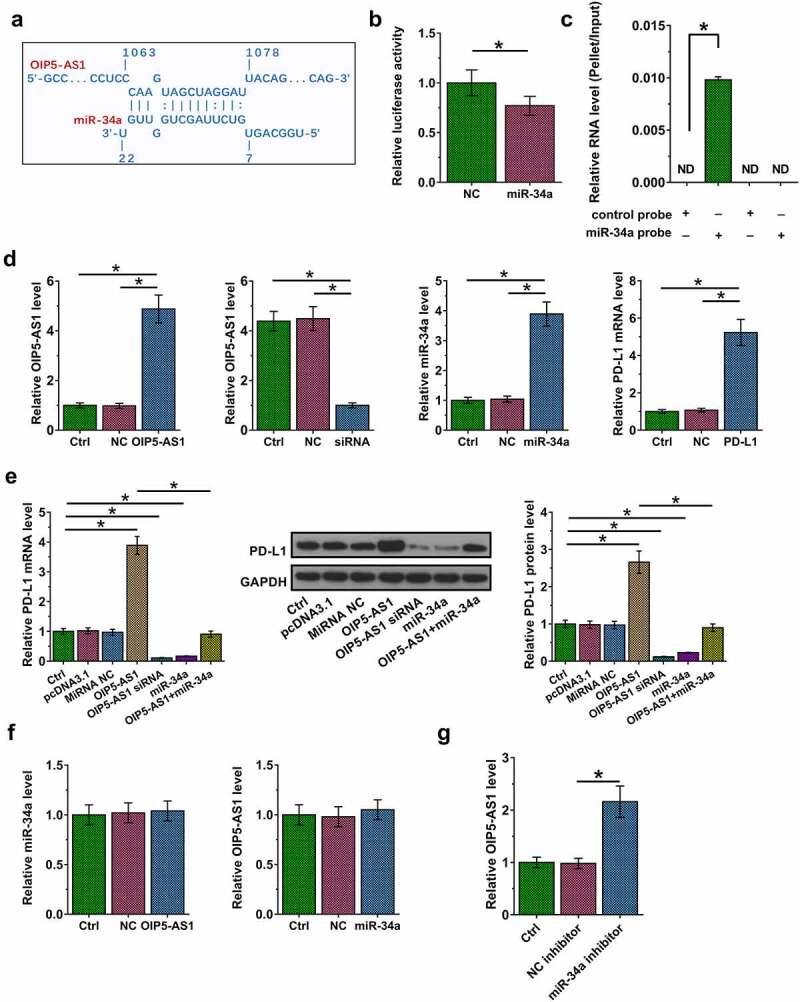
OIP5-AS1 upregulated PD-L1 in H23 cells by targeting miR-34a. The prediction of RNAs interaction was performed by IntaRNA (a). By co-transfecting OIP5-AS1 expression vector plus negative control miRNA (NC group) or miR-34a mimic (miR-34a group) into 10^6^ cells, dual luciferase reporter assay was also conducted (b). The luciferase activity was significantly inhibited in miR-34a group. RNA pull down was conducted (c). OIP5-AS1 cDNA could be only amplified in the precipitation pulled down by miR-34a probe but not control probe. Overexpression of OIP5-AS1, silence of OIP5-AS, overexpression of miR-34a and overexpression of PD-L1 were confirmed by RT-qPCR at 36 h post-transfection (d). Effects of OIP5-AS1 overexpression, silence and miR-34a overexpression on PD-L1 mRNA and protein expression (e). The regulatory relationship between miR-34a and OIP5-AS1 was evaluated by RT-qPCR. Overexpression of OIP5-AS1 significantly inhibited miR-34a expression (f). PD-L1 expression was detected by RT-qPCR (g). The differences between two groups were analyzed by unpaired t-test. *, *p* < 0.05.

RNA interaction suggests function. Therefore, we next performed RNA interaction prediction via IntaRNA to predict the potential binding of miR-34a to OIP5-AS1. It was observed that miR-34a might bind to OIP5-AS1 (Figure 4(a)). Dual luciferase reporter assay showed that OIP5-AS1 plus miR-34a mimic transfection significantly reduced the relative luciferase activity compared to that in NC group, suggesting the direct interaction between them (Figure 4(b), *p* < 0.05). Moreover, RNA pull-down was carried out to further confirm this interaction. It was observed that miR-34a, but not miR-34a (mut), enriched OIP5-AS1 after pulldown, which confirmed our conclusion that OIP5-AS1 could directly interact with miR-34a (Figure 4(c), *p* < 0.05). RT-qPCR analysis showed that the expression levels of miR-34a were lower in NSCLC tissues (Fig. S1, *p* < 0.05). To study the interaction among OIP5-AS1, miR-34a and P-DL1, which is a known target of miR-34a, OIP5-AS1 and miR-34a mimic, PD-L1 expression vector, and OIP5-AS1 siRNA were transfected into H23 cells. Compared to NC and the control group, the expression of OIP5-AS1, miR-34a and PD-L1 were significantly altered after transfection with the overexpression or silencing vector (Figure 4(d), *p* < 0.05). Moreover, overexpression of OIP5-AS1 led to upregulated, while silencing of OIP5-AS1 and overexpression of miR-34a resulted in downregulation of PD-L1, while miR-34a elevation countered the effects of overexpression of OIP5-AS1 in regulating PD-L1 at both RNA and protein levels (Figure 4(e), *p* < 0.05). Moreover, OIP5-AS1 and miR-34a did not regulate the expression of each other, indicating that OIP5-AS1 is unlikely a target of miR-34a (Figure 4(f), *p* < 0.05). Furthermore, the expression levels of PD-L1 were enhanced in H23 cells transfected with miR-34a inhibitor (Figure 4(g), p < 0.05). Therefore, OIP5-AS1 may sponge miR-34a to suppress its role in downregulating PD-L1.

### OIP5-AS1 promoted H23 and H522 cell proliferation through regulating miR-34a and PD-L1

**Figure 5.**
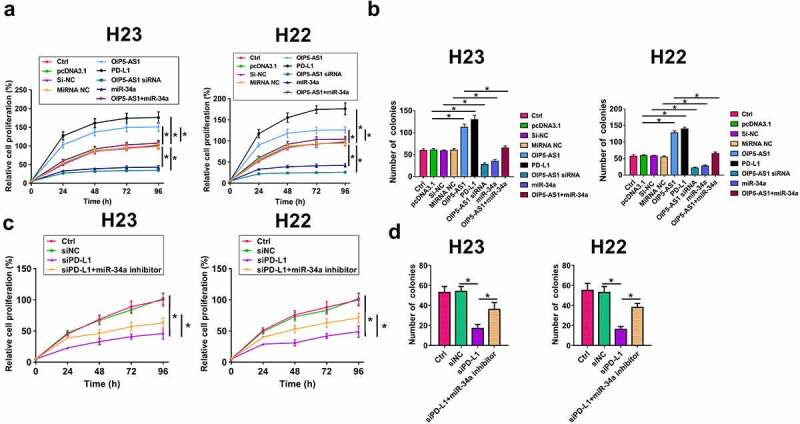
OIP5-AS1 targets miR-34a and regulates PD-L1. Cell proliferation was analyzed by CCK-8 (a) and colony formation assay (b) in H23 and H22 cells. Both overexpression of OIP5-AS1 and PD-L1 significantly promoted cell proliferation, while silencing of OIP5-AS1 and overexpression of miR-34a had opposite effects. To further confirm the roles of above RNAs in cell proliferation, colony formation assay was conducted. Both OIP5-AS1 and PD-L1 overexpression significantly increased cell colony number. However, OIP5-AS1 silence and miR-34a overexpression had opposite effects. The proliferation (c) and colonies (d) was measured by Cell proliferation analysis in H23 and H22 cells transfected with siNC, siPD-L1, and siPD-L1+ miR-34a. *, *p* < 0.05.

Cell proliferation contributes to tumor growth. CCK-8 assay was performed to analyze the role of OIP5-AS1, miR-34a and PD-L1 in cell proliferation. Overexpression of OIP5-AS1 and PD-L1 led to increased proliferation rate and more cell colonies of H23 and H22 cells, while silencing of OIP5-AS1 and overexpression of miR-34a played opposite roles and attenuated the effects of overexpression of OIP5-AS1 (Figure 5(a,b), *p* < 0.05). Moreover, the depletion of PD-L1 inhibited proliferation and colonies of H23 and H22 cells, while miR-34a inhibitor attenuated this effect (Figure 5(c,d), p < 0.05). Therefore, OIP5-AS1 may sponge miR-34a to suppress its role in inhibiting NSCLC cell proliferation.

## Discussion

This study investigated the regulatory relationships among miR-34a, OIP5-AS1 and PD-L1 in NSCLC and found that OIP5-AS1 was upregulated in NSCLC and promoted the expression levels of PD-L1 through targeting miR-34a to promote lung cancer cell proliferation.

The function of OIP5-AS1 has been widely studied in many types of cancer [18–20,25]. One study reported that OIP5-AS1 plays an oncogenic role in cancer biology, and its oncogenic role is achieved through the interactions with different downstream cancer-related factors. OIP5-AS1 is also an oncogene in NSCLC (adenocarcinoma) [18]. Moreover, this study found that OIP5-AS1 was upregulated and could bind to miR-448 to upregulate the expression of Bcl-2, which in turn promoted cancer development [18]. These findings are consistent with our observation that the expression levels of OIP5-AS1 were elevated in NSCLC. Another study reported upregulated expression of OIP5-AS1 in epithelial malignancies [26]. Therefore, we speculated that OIP-AS1 was involved in NSCLC.

PD-L1 is abnormally expressed in multiple cancers [27]. For instance, the expression levels of PD-L1 are decreased in clear cell renal cell carcinoma (CCRCC) tissues and overexpression of PD-L1 promoted CCRCC progression [26]. PD-L1 accelerates breast cancer cell proliferation and migration and inhibits cell apoptosis [27]. In this study, we first observed that the expression levels of PD-L1 were largely elevated in tumor tissues from NSCLC patients. In addition, 5 cases were selected to evaluate PD-L1 expression by immunohistochemistry, which further confirmed the qPCR results. Moreover, we also found that the expression of OIP5-AS1 and PD-L1 were positively correlated in NSCLC tumors but not in non-tumor tissues, indicating that both OIP5-AS1 and PD-L1 may form a crosstalk in the development of NSCLC. To link the pathological stage of NSCLC to the expression of OIP5-AS1 and PD-L1, all 68 patients were classified into 3 stages according to the clinical features and severity. Among them, 16 patients were classified into stage II, 20 patients were sub-grouped into stage III and 32 patients were classified into stage IV. Interestingly, the expression levels of OIP5-AS1 and PD-L1 were both elevated along with disease severity increase, indicating that OIP5-AS1 and PD-L1 may promote the progression of NSCLC [28,29,30].

A previous study reported that OIP5-AS1 acts as a molecular sponge of miR-378a-3p to promote lung cancer cell proliferation and therefore contributes top oor prognosis [21]. Interestingly, our study found that OIP5-AS1 could interact with miR-34a, and its important inhibitory roles has been demonstrated in lung cancer progression [3]. Also, we observed significantly decreased expression levels of miR-34a in NSCLC tumor group (Fig. S1). Therefore, we first predicted their interaction by bioinformatics analysis, which revealed a binding site of miR-34a on OIP5-AS sequence. Next, to confirm our prediction, overexpression of miR-34a significantly reduced the luciferase activity compared to that of the negative control. Moreover, OIP5-AS1 cDNA could only be detected in the combination precipitated by miR-34a probe but not in the control probe and miR-34a (mut) probe. To investigate the regulatory relationships among miR-34a, OIP5-AS1, and PD-L1 we conducted *in vitro* experiments by overexpressing OIP5-AS1, silencing OIP5-AS1 and overexpressing miR-34a in H23 cells. Overexpression of OIP5-AS1 significantly increased the expression levels of PD-L1 at both mRNA and protein levels. On the contrary, silencing of OIP5-AS1 and overexpression of miR-34a induced a decreased expression levels of PD-L1, which was attenuated by miR-34a, suggesting that both OIP5-AS1 and miR-34a could regulate PD-L1 but the roles are opposite. Interestingly, OIP5-AS1 and miR-34a did not regulate the expression of each other. Therefore, OIP5-AS1 is unlikely a target of miR-34a.

Cell proliferation is a promising indicator of tumor pathogenesis. In our study, we conducted CCK-8 and colony formation assay to evaluate the function of OIP5-AS1, miR-34a and PD-L1 in H23 cell proliferation. Interestingly, OIP5-AS1 and PD-L1 both remarkably promoted cell proliferation. However, silencing of OIP5-AS1 and overexpression of miR-34a significantly suppressed H23 cell and H22 cell proliferation. To find more evidence supporting our findings obtained in CCK-8 experiments, we also conducted cell colony assay, which showed consistent results with CCK-8 and revealed that OIP5-AS1 and PD-L1 played negative roles but miR-34a had inhibited effects in the pathogenesis of NSCLC.

Several limitations in this study should be acknowledged. Firstly, other possible mechanisms responsible for OIP5-AS1-mediated NSCLC progression remain to be investigated in the future. Secondly, the clinical sample size in this study can be enlarged. Finally, *in vivo* animal assays should be performed to test the function of OIP5-AS1/miR-34a/PD-L1 axis. Although the involvement of lncRNAs in NSCLC has been reported [31,32–36], the role of most lncRNAs in this cancer is still unclear and more studies are needed.

## Conclusion

Overall, this study characterized a novel OIP5-AS1/miR-34a/PD-L1 axis involved in NSCLC progression. As miR-34a participates in the inhibition of many types of cancer, the initiation of upstream regulators of miR-34a may be considered as a novel strategy to interfere the pathogenesis of NSCLC and to develop a novel therapeutic approach for NSCLC.

## Supplementary Material

Supplemental Material

## Data Availability

The analyzed data sets generated during the study are available from the corresponding author on reasonable request.

## References

[1] Sung H, Ferlay J, Siegel RL, et al. Global cancer statistics 2020: GLOBOCAN estimates of incidence and mortality worldwide for 36 cancers in 185 countries. CA Cancer J Clin. 2021 Feb;71(3):209–249. 33538338 10.3322/caac.21660

[2] Siegel RL, Miller KD, Fuchs HE, et al. Cancer statistics, 2021. CA Cancer J Clin. 2021 Jan;71(1):7–33. 33433946 10.3322/caac.21654

[3] Jung KW, Won YJ, Kong HJ, et al. Cancer statistics in Korea: incidence, mortality, survival, and prevalence in 2015. Cancer Res Treat. 2018 Apr;50(2):303–316. 29566481 10.4143/crt.2018.143 PMC5912151

[4] O’Keeffe LM, Taylor G. Smoking as a risk factor for lung cancer in women and men: a systematic review and meta-analysis. BMJ open. 2018 Oct 3;8(10):e021611. 10.1136/bmjopen-2018-021611 PMC6194454 30287668

[5] Stiles BM, Rahouma M, Hussein MK, et al. Never smokers with resected lung cancer: different demographics, similar survival. Eur J Cardiothorac Surg. 2018 Apr 1;53(4):842–848. 29182735 10.1093/ejcts/ezx390

[6] Suda K, Mitsudomi T. Racial differences in lung cancer genetics. J Thorac Oncol. 2015 Feb;10(2):230–231. 25611225 10.1097/JTO.0000000000000439

[7] Kerr KM, Tsao MS, Nicholson AG, et al. Programmed death-ligand 1 immunohistochemistry in lung cancer: in what state is this art? J Thorac Oncol. 2015 Jul;10(7):985–989. 26134220 10.1097/JTO.0000000000000526

[8] Fan Y, Che X, Hou K, et al. MiR-940 promotes the proliferation and migration of gastric cancer cells through up-regulation of programmed death ligand-1 expression. Exp Cell Res. 2018 Dec 15;373(1–2):180–187. 30367831 10.1016/j.yexcr.2018.10.011

[9] Wang X, Li J, Dong K, et al. Tumor suppressor miR-34a targets PD-L1 and functions as a potential immunotherapeutic target in acute myeloid leukemia. Cell Signal. 2015 Mar;27(3):443–452. 25499621 10.1016/j.cellsig.2014.12.003

[10] Dong J, Zhu D, Tang X, et al. Circulating tumor cells in pulmonary vein and peripheral arterial provide a metric for PD-L1 diagnosis and prognosis of patients with non-small cell lung cancer. PloS one. 2019;14(7):e0220306. 31348821 10.1371/journal.pone.0220306 PMC6660086

[11] Li H, Xu Y, Wan B, et al. The clinicopathological and prognostic significance of PD-L1 expression assessed by immunohistochemistry in lung cancer: a meta-analysis of 50 studies with 11,383 patients. Transl Lung Cancer Res. 2019 Aug;8(4):429–449. 31555517 10.21037/tlcr.2019.08.04 PMC6749117

[12] Yang H, Shi J, Lin D, et al. Prognostic value of PD-L1 expression in combination with CD8(+) TILs density in patients with surgically resected non-small cell lung cancer. Cancer Med. 2018 Jan;7(1):32–45. 29168339 10.1002/cam4.1243 PMC5773962

[13] Paydas S, Bağır E, Seydaoglu G, et al. Programmed death-1 (PD-1), programmed death-ligand 1 (PD-L1), and EBV-encoded RNA (EBER) expression in Hodgkin lymphoma. Ann Hematol. 2015 Sep;94(9):1545–1552. 26004934 10.1007/s00277-015-2403-2

[14] Fatica A, Bozzoni I. Long non-coding RNAs: new players in cell differentiation and development. Nat Rev Genet. 2014 Jan;15(1):7–21. 24296535 10.1038/nrg3606

[15] Huarte M. The emerging role of lncRNAs in cancer. Nat Med. 2015 Nov;21(11):1253–1261. 26540387 10.1038/nm.3981

[16] Ramilowski JA, Chi WY, Agrawal S, et al. Functional annotation of human long non-coding RNAs via molecular phenotyping. 2020 Jul;30(7):1060-1072. 10.1101/gr.254219.119 PMC7397864 32718982

[17] Paraskevopoulou MD, Hatzigeorgiou AG. Analyzing MiRNA-LncRNA Interactions. Methods Mol Biol. 2016;1402:271–286. 26721498 10.1007/978-1-4939-3378-5_21

[18] Deng J, Deng H, Liu C, et al. Long non-coding RNA OIP5-AS1 functions as an oncogene in lung adenocarcinoma through targeting miR-448/Bcl-2. Biomed Pharmacother. 2018 Feb;98:102–110. 29247949 10.1016/j.biopha.2017.12.031

[19] Wang Y, Shi F, Xia Y, et al. LncRNA OIP5-AS1 predicts poor prognosis and regulates cell proliferation and apoptosis in bladder cancer. J Cell Biochem. 2019;120:7499–7505. 10.1002/jcb.28024 30485498

[20] Zou Y, Yao S, Chen X, et al. LncRNA OIP5-AS1 regulates radioresistance by targeting DYRK1A through miR-369-3p in colorectal cancer cells. Eur J Cell Biol. 2018 Jun;97(5):369–378. 29773344 10.1016/j.ejcb.2018.04.005

[21] Wang M, Sun X, Yang Y, et al. Long non-coding RNA OIP5-AS1 promotes proliferation of lung cancer cells and leads to poor prognosis by targeting miR-378a-3p. Thorac Cancer. 2018 Aug;9(8):939–949. 29897167 10.1111/1759-7714.12767 PMC6068457

[22] Han Z, Zhang Y, Yang Q, et al. miR-497 and miR-34a retard lung cancer growth by co-inhibiting cyclin E1 (CCNE1). Oncotarget. 2015 May 30;6(15):13149–13163. 25909221 10.18632/oncotarget.3693 PMC4537005

[23] Grentzmann G, Ingram JA, Kelly PJ, et al. A dual-luciferase reporter system for studying recoding signals. RNA. 1998 Apr;4(4):479–486. 9630253 PMC1369633

[24] Livak KJ, Schmittgen TD. Analysis of relative gene expression data using real-time quantitative PCR and the 2− ΔΔCT method. Methods. 2001 Dec;25(4):402–408. 11846609 10.1006/meth.2001.1262

[25] Yang N, Chen J, Zhang H, et al. LncRNA OIP5-AS1 loss-induced microRNA-410 accumulation regulates cell proliferation and apoptosis by targeting KLF10 via activating PTEN/PI3K/AKT pathway in multiple myeloma. Cell Death Dis. 2017 Aug 10;8(8):e2975. 28796257 10.1038/cddis.2017.358 PMC5596549

[26] Arunkumar G, Anand S, Raksha P, et al. LncRNA OIP5-AS1 is overexpressed in undifferentiated oral tumors and integrated analysis identifies as a downstream effector of stemness-associated transcription factors. Sci Rep. 2018 May 4;8(1):7018. 29728583 10.1038/s41598-018-25451-3 PMC5935738

[27] Chang K, Qu Y, Dai B, et al. PD-L1 expression in Xp11.2 translocation renal cell carcinoma: indicator of tumor aggressiveness. Sci Rep. 2017 May 18;7(1):2074. 28522811 10.1038/s41598-017-02005-7 PMC5437001

[28] Azarbarzin S, Hosseinpour-Feizi MA, Banan Khojasteh SM, et al. MicroRNA −383-5p restrains the proliferation and migration of breast cancer cells and promotes apoptosis via inhibition of PD-L1. Life Sci. 2021 Feb 15;267:118939. 33359245 10.1016/j.lfs.2020.118939

[29] Cui Y, Yang X, Zhang X. Shrimp miR-34 from shrimp stress response to virus infection suppresses tumorigenesis of breast cancer. Mol Ther Nucleic Acids. 2017 Dec 15;9:387–398. 29246317 10.1016/j.omtn.2017.10.016 PMC5694971

[30] Ji Q, Hao X, Meng Y, et al. Restoration of tumor suppressor miR-34 inhibits human p53-mutant gastric cancer tumorspheres. BMC Cancer. 2008 Sep 21;8(1):266. 18803879 10.1186/1471-2407-8-266 PMC2564978

[31] Shen D, Li J, Tao K, et al. Long non-coding RNA MCM3AP antisense RNA 1 promotes non-small cell lung cancer progression through targeting microRNA-195-5p. Bioengineered. 2021 Aug;12(1):3525–3538. 34346845 10.1080/21655979.2021.1950282 PMC8806479

[32] Feng J, Li J, Qie P, et al. [Long non-coding RNA (lncRNA) PGM5P4-AS1 inhibits lung cancer progression by up-regulating leucine zipper tumor suppressor (LZTS3) through sponging microRNA miR-1275]. Bioengineered. 2021 Dec;12(1):196–207. 33315502 10.1080/21655979.2020.1860492 PMC8806334

[33] Ma Y, Bao-Han W, Lv X, et al. MicroRNA-34a mediates the autocrine signaling of PAR2-activating proteinase and its role in colonic cancer cell proliferation. PloS one. 2013;8(8):e72383. 23991105 10.1371/journal.pone.0072383 PMC3753253

[34] Kim JS, Kim EJ, Lee S, et al. MiR-34a and miR-34b/c have distinct effects on the suppression of lung adenocarcinomas. Exp Mol Med. 2019 Jan 17;51(1):1–10. 10.1038/s12276-018-0203-1 PMC6353903 30700696

[35] Qu F, Ye J, Pan X, et al. MicroRNA-497-5p down-regulation increases PD-L1 expression in clear cell renal cell carcinoma. J Drug Target. 2019 Jan;27(1):67–74. 30183478 10.1080/1061186X.2018.1479755

[36] Wang F, Gu T, Chen Y, et al. Long non-coding RNA SOX21-AS1 modulates lung cancer progress upon microRNA miR-24-3p/PIM2 axis. Bioengineered. 2021 Sep;12(1):6724–6737. 34511042 10.1080/21655979.2021.1955578 PMC8806559

