# Supplementary figures and images for: Long non-coding RNA Opa interacting protein 5-antisense RNA 1 binds to micorRNA-34a to upregulate oncogenic PD-L1 in non-small cell lung cancer
Source: Bioengineered. 2022 Apr 12;13(4):9264–73. doi: 10.1080/21655979.2022.2036904 (PMC9161958; doi:10.1080/21655979.2022.2036904)

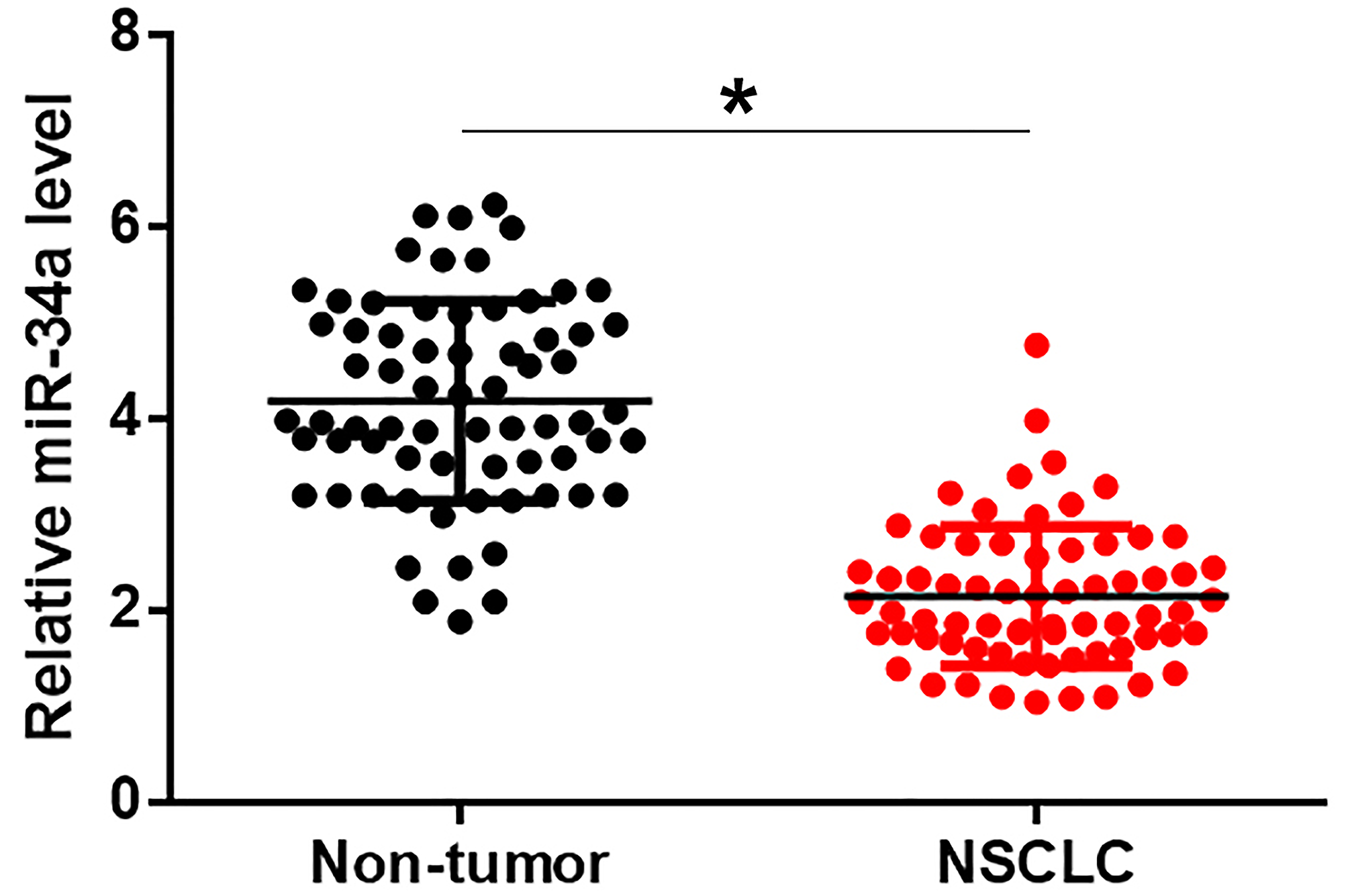

Supplement: Supplemental Material [file KBIE_A_2036904_SM0744.tif]
